# Evaluation of the MEFIER Score in Identifying Patients at High Risk of Endocarditis in *Enterococcus faecalis* Bacteremia

**DOI:** 10.1093/ofid/ofag184

**Published:** 2026-03-28

**Authors:** Virgile Zimmermann, Nicolas Fourré, Pierre Monney, Georgios Tzimas, Laurence Senn, Lars Niclauss, Matthias Kirsch, Benoit Guery, Matthaios Papadimitriou-Olivgeris

**Affiliations:** Infectious Diseases Service, Lausanne University Hospital and University of Lausanne, Lausanne, Switzerland; Infectious Diseases Service, Lausanne University Hospital and University of Lausanne, Lausanne, Switzerland; Department of Cardiology, Lausanne University Hospital and University of Lausanne, Lausanne, Switzerland; Department of Cardiology, Lausanne University Hospital and University of Lausanne, Lausanne, Switzerland; Infectious Diseases Service, Lausanne University Hospital and University of Lausanne, Lausanne, Switzerland; Infection Prevention and Control Unit, Lausanne University Hospital and University of Lausanne, Lausanne, Switzerland; Department of Cardiac Surgery, Lausanne University Hospital and University of Lausanne, Lausanne, Switzerland; Department of Cardiac Surgery, Lausanne University Hospital and University of Lausanne, Lausanne, Switzerland; Infectious Diseases Service, Lausanne University Hospital and University of Lausanne, Lausanne, Switzerland; Infectious Diseases Service, Lausanne University Hospital and University of Lausanne, Lausanne, Switzerland; Infectious Diseases Service, Hospital of Valais and Institut Central des Hôpitaux, Sion, Switzerland

**Keywords:** bacteremia, *Enterococcus faecalis*, infective endocarditis, NOVA score, predictive score

## Abstract

In a retrospective cohort of 549 *Enterococcus faecalis* bacteremia episodes, the MEFIER score showed poor rule-out performance (negative likelihood ratio of 0.69), misclassifying 60% of IE episodes as low-risk. In contrast, DENOVi score achieved a low negative likelihood ratio (0.07), misclassifying only 5% while limiting unnecessary echocardiography.

## Teaser

Among 549 *Enterococcus faecalis* bacteremia episodes, the MEFIER score failed to reliably rule out infective endocarditis, misclassifying 60% of cases as low risk, whereas DENOVi demonstrated superior rule-out performance, misclassifying only 5% while reducing echocardiography use.

*Enterococcus faecalis* is recognized as one of the leading pathogens responsible for infective endocarditis (IE), with both the 2023 International Society of Cardiovascular Infectious Diseases (ISCVID) and European Society of Cardiology (ESC) Duke criteria classifying *E. faecalis* as a typical microorganism for IE [1–4]. Furthermore, the 2023 ESC guidelines recommend echocardiographic evaluation for all patients with *E. faecalis* bacteremia (EfsB) [4]. Several risk scores have been developed to stratify the likelihood of IE in patients with EfsB, including the NOVA, DENOVA, and DENOVi scores, which are based on clinical characteristics [5–10]. However, a recently developed score, the machine learning-derived *E. faecalis* Infective Endocarditis Risk (MEFIER) score, derived from a large cohort, is unique in relying exclusively on objective parameters available at the time of bacteremia, enabling automated calculation directly from electronic health records [11].

This study aimed to evaluate the performance of the MEFIER score and other existing scores in predicting IE among patients with EfsB.

## METHODS

This retrospective study was conducted at Lausanne University Hospital and included patients from the institutional bacteremia cohort between January 2015 and December 2024. The study was approved by the Ethics Committee of the Canton of Vaud (CER-VD 2021-02516).

Inclusion criteria were adult patients (≥18 years of age) with at least 1 blood culture positive for *E. faecalis* and no documented refusal of data use. Exclusion criteria included patients with incomplete medical records, such as those transferred to other hospitals at the onset of infection without available follow-up data.

Data on demographics, clinical presentation, imaging, microbiology, surgery, and pathology were extracted from patients’ electronic health records. Infectious diseases (ID) consultants were notified of all positive blood cultures once the causative organism was identified. Although ID consultation for EfsB was not mandatory, it was provided at the discretion of the attending physician [12].

The date of the first positive blood culture was defined as the onset of infection. A new EfsB episode was recorded if more than 30 days had elapsed since completion of antimicrobial therapy for a previous episode. Bacteremia episodes were classified as community-acquired, healthcare-associated, or nosocomial according to the criteria established by Friedman et al [13]. The site of infection was determined by the ID consultant based on a comprehensive assessment of clinical, radiological, microbiological, and surgical findings.

The primary outcome was the negative likelihood ratio (NLR), which was used as a measure of the ability of each score to safely rule out IE. Published score thresholds were applied: MEFIER (≥32 points) [11], adapted NOVA (≥4 points) [8], DENOVA (≥3 points) [6], and DENOVi (≥2 points) [10] (Supplementary Table 1). Two reference standards were used: (1) the diagnosis established by the Endocarditis Team (since January 2018) or, prior to that date, by expert clinicians (M. P. O., P. M.) who fulfilled the role of IE adjudicators, and (2) the definite IE classification based on the 2023 ISCVID Duke criteria [2].

Statistical analyses were performed using SPSS version 26.0 (SPSS, Chicago, IL, USA). Categorical variables were compared using the *χ*² test or Fisher's exact test, as appropriate, and continuous variables were analyzed using the Mann–Whitney *U* test. The performance of each score in identifying episodes at high risk for IE was assessed by measuring agreement between the score-based risk classification and each reference standard. Sensitivity, specificity, positive and negative predictive values (PPV and NPV), positive likelihood ratio (PLR), NLR, and overall accuracy were calculated, each with corresponding 95% CIs. All statistical tests were 2-tailed, and a *P*-value <.05 was considered statistically significant.

## RESULTS

A total of 549 EfsB episodes were included, occurring in 519 patients (Supplementary Figure 1). Cardiac imaging was performed in 318 (58%) episodes. ID consultation was provided in 441 (80%) episodes. Follow-up blood cultures documenting clearance were obtained in 445 (81%) episodes. Overall, 499 (91%) episodes had adequate diagnostic assessment, defined as cardiac imaging, valve surgery, or autopsy (Supplementary Table 2).

IE was diagnosed in 115 (21%) episodes by the Endocarditis Team, of which 80 (70%) were classified as definite IE and 35 (30%) as possible IE according to the 2023 ISCVID-Duke criteria (Supplementary Table 3). Recurrence of EfsB within 90 days of the initial episode occurred in 12 (2%) episodes (Supplementary Table 4). Among the 434 EfsB episodes without IE, 2 (0.4%) developed *E. faecalis* IE within 90 days of the initial episode. Ninety-day mortality was 26% (142 episodes).

The MEFIER score classified 105 (19%) episodes as high risk for IE, corresponding to an absolute reduction of 39% in the proportion of echocardiographic examinations compared with the 58% performed based on clinical evaluation alone (Table 1). Using Endocarditis Team or expert clinicians as the reference standard, 69 (60%) IE episodes were incorrectly classified as low risk, resulting in a NLR of 0.69 (95% CI: 0.60–0.81) (Table 2).

**Table 1.** Classifications based on Different Predictive Scores Among 549 Episodes With *E. faecalis* Bacteremia, Using the Endocarditis Team or Expert Clinician Adjudication to Determine the Presence or Absence of Infective Endocarditis

	No Infective Endocarditis (n = 434)	Infective Endocarditis (n = 115)	*P*
**MEFIER score**			
Age			.219
<43 y (3 points), n (%)	23 (5)	9 (8)	
≥43 to <65 y (6 points), n (%)	109 (25)	21 (18)	
≥65 y (0 points), n (%)	302 (70)	85 (74)	
Male sex (2 points), n (%)	309 (71)	85 (74)	.641
Prior history of IE (6 points), n (%)	7 (2)	16 (14)	<.001
Valvular heart disease (28 points), n (%)	30 (7)	25 (22)	<.001
Congenital heart disease (14 points), n (%)	5 (1)	8 (7)	.001
Cardiac implantable electronic device (12 points), n (%)	32 (7)	28 (24)	<.001
Non-nosocomial onset (11 points), n (%)	173 (40)	90 (78)	<.001
Abnormal laboratory values			
Platelet count (1 point), n (%)	159 (37)	32 (28)	.079
Albumin (3 points), n (%)	180 (42)	26 (23)	<.001
Hemoglobin (16 points), n (%)	295 (68)	78 (68)	1.000
MEFIER score (points), median (IQR)	21 (16–28)	30 (19–36)	<.001
High MEFIER score (≥32 points), n (%)	59 (14)	46 (40)	<.001
**Adapted NOVA score**			
Number of positive blood cultures (N; +5 points), n (%)	211 (49)	106 (92)	<.001
Unknown origin of bacteremia (O; 4 points), n (%)	128 (30)	112 (97)	<.001
Valve disease (V; +2 points), n (%)	34 (8)	61 (53)	<.001
Auscultation of a heart murmur (A; +1 point), n (%)	60 (14)	77 (68)	<.001
Adapted NOVA score (points), median (IQR)	5 (0–5)	11 (10–12)	<.001
High adapted NOVA score (≥4 points), n (%)	274 (63)	114 (99)	<.001
**DENOVA score**			
Duration of symptoms ≥7 d (D; +1 point), n (%)	18 (4)	65 (57)	<.001
Embolization (E; +1 point), n (%)	8 (2)	45 (39)	<.001
Number of positive cultures ≥2 (N; +1 point), n (%)	211 (49)	106 (92)	<.001
Origin of infection unknown (O; +1 point), n (%)	128 (30)	112 (97)	<.001
Valve disease (V; +1 point), n (%)	34 (8)	61 (53)	<.001
Auscultation of murmur (A; +1 point), n (%)	60 (14)	77 (68)	<.001
DENOVA score (points), median (IQR)	1 (0–2)	4 (3–5)	<.001
High DENOVA score (≥3 points), n (%)	30 (7)	104 (90)	<.001
**DENOVi score**			
Valve disease, intracardiac prosthetic material (Vi; +1 point), n (%)	62 (14)	69 (60)	<.001
DENOVi score (points), median (IQR)	1 (0–1)	4 (3–4)	<.001
High DENOVi score (≥2 points), n (%)	105 (24)	109 (95)	<.001

Abbreviation: IQR, interquartile range.

**Table 2.** Performance of Different Predictive Scores in Identifying Patients at High-risk for Infective Endocarditis Among All 549 Episodes of *E. faecalis* Bacteremia or the Subgroup of 499 Episodes With Adequate Cardiac Imaging, Cardiac Surgery, or Autopsy

	High Risk N (%)	Sensitivity % (95% CI)	Specificity % (95% CI)	PPV % (95% CI)	NPV % (95% CI)	PLR % (95% CI)	NLR % (95% CI)	Accuracy % (95% CI)
**Reference standard: Endocarditis Team or expert clinicians; whole cohort: 549 episodes**								
MEFIER	105 (19)	40 (31–50)	86 (83–89)	44 (36–52)	84 (82–86)	2.94 (2.12–4.08)	0.69 (0.60–0.81)	77 (73–80)
Adapted NOVA	388 (71)	99 (96–100)	37 (32–42)	29 (28–31)	99 (96–100)	1.57 (1.46–1.69)	0.02 (0.00–0.17)	50 (46–54)
DENOVA	134 (24)	90 (84–95)	93 (90–95)	78 (71–83)	97 (95–98)	13.8 (9.22–18.6)	0.10 (0.06–0.18)	93 (90–95)
DENOVi	214 (39)	95 (89–98)	76 (71–80)	51 (47–55)	98 (96–99)	3.92 (3.30–4.65)	0.07 (0.03–0.15)	80 (76–83)
**Reference standard: Endocarditis Team or expert clinicians; adequate cardiac imaging, valve surgery, or autopsy: 499 episodes**								
MEFIER	90 (18)	42 (32–53)	85 (84–91)	43 (35–52)	87 (85–89)	3.34 (2.35–4.74)	0.66 (0.56–0.79)	79 (75–82)
Adapted NOVA	344 (69)	99 (94–100)	38 (33–43)	27 (25–28)	99 (96–100)	1.60 (1.48–1.73)	0.03 (0.00–0.20)	50 (45–54)
DENOVA	109 (22)	92 (85–97)	95 (92–96)	79 (71–85)	98 (96–99)	16.3 (10.9–24.4)	0.08 (0.04–0.16)	94 (92–96)
DENOVi	177 (36)	96 (89–99)	78 (67–76)	50 (46–55)	99 (97–100)	4.42 (3.65–5.34)	0.05 (0.02–0.14)	82 (78–85)
**Reference standard: definite infective endocarditis based on 2023 ISCVID Duke criteria; whole cohort: 549 episodes**								
MEFIER	105 (19)	41 (30–53)	85 (81–88)	31 (25–39)	89 (88–91)	2.69 (1.92–3.76)	0.69 (0.58–0.84)	78 (75–82)
Adapted NOVA	388 (71)	100 (95–100)	34 (30–39)	21 (20–22)	100 (98–100)	1.52 (1.43–1.63)	0.02 (0.00–0.29)	44 (40–48)
DENOVA	134 (24)	95 (88–99)	88 (84–90)	57 (51–63)	99 (98–100)	7.68 (6.01–9.83)	0.06 (0.02–0.15)	89 (86–91)
DENOVi	214 (39)	98 (91–100)	71 (67–76)	36 (33–40)	99 (98–100)	3.36 (2.91–3.89)	0.04 (0.01–0.14)	75 (71–78)

Abbreviations: CI, confidence interval; ISCVID, International Society of Cardiovascular Infectious Diseases; NLR, negative likelihood ratio; NPV, negative predictive value; PLR, positive likelihood ratio; PPV, positive predictive value.

The adapted NOVA, DENOVA, and DENOVi scores classified 388 (71%), 134 (24%), and 214 (39%) episodes, respectively, as high risk for IE (Table 1). Compared with clinical practice, the adapted NOVA score resulted in an absolute increase of 13% in the proportion of echocardiographic examinations, whereas DENOVA and DENOVi resulted in absolute reductions of 34% and 19%, respectively. The corresponding NLRs were 0.02 (95% CI: 0.00–0.17) for adapted NOVA, 0.10 (95% CI: 0.06–0.18) for DENOVA, and 0.07 (95% CI: 0.03–0.15) for DENOVi (Table 2).

## DISCUSSION

This study is the first to externally validate the MEFIER score for predicting IE among patients with EfsB and to comprehensively compare its performance with other available predictive tools. Our findings demonstrate that the MEFIER score failed to identify the majority of IE episodes.

Specifically, the MEFIER score misclassified 69 (60%) IE episodes as low risk, resulting in an NPV of 84% and a NLR of 0.69; values that are insufficient to safely rule out IE [11]. These results contrast sharply with those of the original derivation study, in which the MEFIER score achieved an NPV of 98%. Several factors may explain this discrepancy. First, in the original study, the diagnosis of IE, comorbidities, and prior procedures relied on International Classification of Diseases (ICD) codes, a methodology previously shown to be associated with a high risk of misclassification [11]. This likely contributed to the unusually low reported incidence of IE, with only 86 of 2535 patients (3%) diagnosed with IE [11, 14]. In contrast, prior studies of EfsB have reported IE incidences ranging from 4% to 26%, with most estimates clustering around 15% [6, 8, 15–17]. The low incidence observed in the MEFIER derivation cohort is therefore likely attributable to misclassification related to ICD-based case identification [11, 14]. Moreover, the derivation study did not report the proportion of patients who underwent cardiac imaging. In a separate study using the same database among patients with *Staphylococcus aureus* bacteremia, only 3% underwent echocardiography (transthoracic or transesophageal), further suggesting substantial limitations of approaches relying exclusively on administrative coding [14, 18].

From a practical standpoint, the MEFIER score incorporates numerous variables with weighted point assignments, limiting its usability at the bedside [11]. In addition, several well-established predictors of IE, such as embolic events, unknown source of bacteremia, and duration of symptoms, are not included. Although the score was designed to allow early calculation at the time of blood culture positivity using readily available objective data, it did not fulfill its primary objective of safely ruling out IE, as it failed to identify most IE cases [11].

In contrast, all comparator scores (the adapted NOVA, DENOVA, and DENOVi) demonstrated strong rule-out performance, with NLRs ≤ 0.10. Such values support their use in excluding IE when applied in conjunction with clinical judgment. However, consistent with prior studies, the adapted NOVA score substantially overestimated IE risk, classifying 71% of episodes as high risk and resulting in a 13% absolute increase in the need for cardiac imaging compared with clinical practice alone (58%) [5, 7, 8, 10]. By contrast, both DENOVA and DENOVi were associated with meaningful reductions in echocardiography utilization [6, 7, 9, 10, 19].

This study has several limitations. First, its retrospective, single-center design limits generalizability. Second, using the Endocarditis Team's assessment as the reference standard may introduce misclassification bias; however, it reflects guideline-recommended multidisciplinary practice [4, 20]. To further mitigate this limitation, we also applied the 2023 ISCVID-Duke criteria as an independent parallel reference standard. Third, 42% of patients did not undergo cardiac imaging. However, most of these patients received short courses of antimicrobial therapy (<14 days) and only 0.4% developed IE during 90 days of follow-up, suggesting that misclassification among non-IE episodes was uncommon and unlikely to have materially influenced the results. Fourth, 426 out of the 549 (78%) EfsB episodes included in this study were part of the 827 enterococcal bacteremia episodes used to derive the DENOVi score, which may partly explain its favorable performance in our cohort [10]. However, the score was originally developed to assess the risk of IE among bacteremia caused by any *Enterococcus* spp. and was not specifically evaluated in the subgroup of EfsB.

In summary, the MEFIER score failed to identify most IE cases as high risk, rendering it unsuitable for clinical application in patients with EfsB. In contrast, the adapted NOVA, DENOVA, and DENOVi scores demonstrated strong rule-out capability, although the adapted NOVA score markedly overestimated risk and increased imaging burden. Among the latter two, DENOVi appeared to offer the most balanced diagnostic performance, minimizing missed IE cases while avoiding unnecessary echocardiography. Further external validation is warranted to determine which prediction tool is best suited for use alongside clinical judgment to optimize resource utilization and diagnostic accuracy in patients with EfsB.

## Supplementary Material

ofag184_Supplementary_Data

## Supplementary Data

Supplementary materials are available at *Open Forum Infectious Diseases* online. Consisting of data provided by the authors to benefit the reader, the posted materials are not copyedited and are the sole responsibility of the authors, so questions or comments should be addressed to the corresponding author.
