# Supplementary material for: Evaluation of the MEFIER Score in Identifying Patients at High Risk of Endocarditis in Enterococcus faecalis Bacteremia
Source: Open Forum Infect Dis. 2026 Mar 28;13(4):ofag184. doi: 10.1093/ofid/ofag184 (PMC13061135; doi:10.1093/ofid/ofag184)
Supplement: ofag184_Supplementary_Data [file ofag184_supplementary_data.docx]

**Supplementary Figure 1.** Flowchart of included patients

**
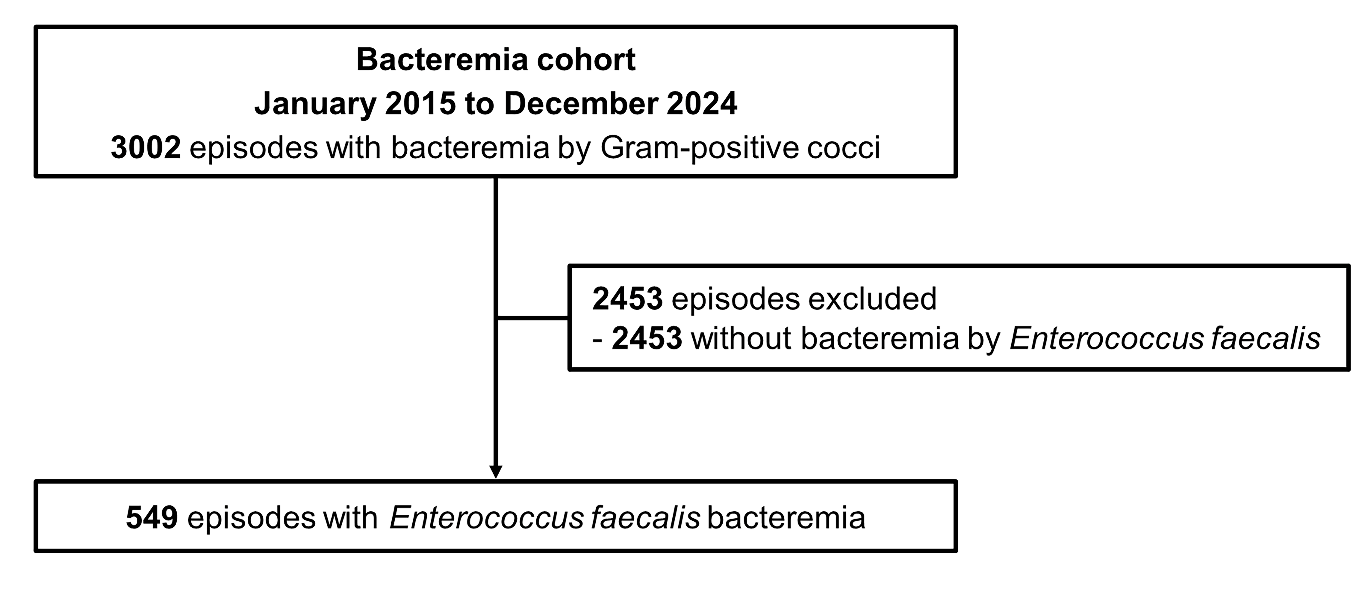
**

**Supplementary Table 1.** Overview of adapted NOVA, DENOVA and DENOVI scores

| **MEFIER score** |  | **Adapted NOVA score** |  | **DENOVA score** |  | **DENOVi score** |  |
| --- | --- | --- | --- | --- | --- | --- | --- |
| **Items** | **Points** | **Items** | **Points** | **Items** | **Points** | **Items** | **Points** |
| Age |  |  |  | **D:** Duration of symptoms ≥7 days | 1 | **D:** Duration of symptoms ≥7 days | 1 |
| <43 years | 3 |  |  | **E:** Embolization | 1 | **E:** Embolization | 1 |
| ≥43 to <65 years | 6 | **N:** Number of positive blood cultures^a^ | 5 | **N:** Number of positive blood cultures^a^ | 1 | **N:** Number of positive blood cultures^a^ | 1 |
| ≥65 years | 0 | **O:** Unknown origin of bacteremia | 4 | **O:** Unknown origin of bacteremia | 1 | **O:** Unknown origin of bacteremia | 1 |
| Male sex | 2 | **V:** Valve disease^b^ | 2 | **V:** Valve disease^b^ | 1 | **Vi:** Valve disease^b^, intracardiac electronic devices^c^ | 1 |
| Prior history of IE | 6 | **A:** Auscultation of a heart murmur | 1 | **A:** Auscultation of a heart murmur | 1 |  |  |
| Valvular heart disease | 28 |  |  |  |  |  |  |
| Congenital heart disease | 14 |  |  |  |  |  |  |
| Cardiac implantable electronic device | 12 |  |  |  |  |  |  |
| Non-nosocomial osent | 11 |  |  |  |  |  |  |
| Abnormal laboratory values |  |  |  |  |  |  |  |
| Platelet count | 1 |  |  |  |  |  |  |
| Albumin | 3 |  |  |  |  |  |  |
| Hemoglobin | 16 |  |  |  |  |  |  |
| Cutoff: ≥32 points |  | Cutoff: ≥4 points |  | Cutoff: ≥3 points |  | Cutoff: ≥2 points |  |
|  |  |  |  |  |  |  |  |

^a^Number of positive blood cultures refers to two out of two positive blood culture sets, three out of three positive sets, or the majority of sets if more than three were drawn.

^b^Valve disease refers to native valve disease (classified as moderate or high risk for infective endocarditis), previous infective endocarditis, or the presence of a prosthetic valve.

^b^Intracardiac electronic devices refer to cardiac implantable electronic devices

**Supplementary Table 2.** Definitions of adequate cardiac imaging according to different clinical contexts

| **Clinical context** | **Cardiac imaging** |
| --- | --- |
| Presence of vegetation, perforation, abscess, aneurysm, pseudoaneurysm, or fistula | TTE, TEE, or cardiac CT |
| Abnormal metabolic activity on involving a valve or CIED lead | [^18^F]FDG-PET/CT |
| Presence of prosthetic valves | [^18^F]FDG-PET/CT or cardiac CT |
| Presence of a CIED without a prosthetic valve | TEE |
| Absence of prosthetic intracardiac material |  |
| - At least two factors: community acquired, unknown focus, persistent bacteremia, embolic events | TEE or cardiac CT |
| - Monomicrobial bacteremia and one aforementioned factor | TEE or cardiac CT |
| - Polymicrobial bacteremia and one aforementioned factor | TTE |
| - None of aforementioned factors | No exam |

[^18^F]FDG-PET/CT: [^18^F]fluorodeoxyglucose positron emission tomography/computed tomography; CIED: cardiac implantable electronic device; TEE: transesophageal echocardiography; TTE transthoracic echocardiography

**Supplementary Table 3.** Comparison of episodes with or without infective endocarditis diagnosis among 549 episodes with *E. feacalis* bacteremia

|  | **No infective endocarditis (n=434)** | **Infective endocarditis (n=115)** | ***P*** |
| --- | --- | --- | --- |
| Demographics |  |  |  |
| Male sex, n (%) | 309 (71) | 85 (74) | 0.641 |
| Age (years), median (IQR) | 71 (62-80) | 75 (63-82) | 0.133 |
| Cardiac predisposing factors |  |  |  |
| Intravenous drug use, n (%) | 7 (2) | 9 (8) | 0.002 |
| Congenital disease, n (%) | 5 (1) | 8 (7) | 0.001 |
| Moderate or severe valve regurgitation/stenosis, n (%) | 27 (7) | 43 (26) | <0.001 |
| Prior endocarditis, n (%) | 7 (2) | 16 (14) | <0.001 |
| Surgical prosthetic valve/transcatheter aortic valve replacement, n (%) | 26 (6) | 50 (44) | <0.001 |
| Cardiac implantable electronic devices, n (%) | 32 (7) | 28 (24) | <0.001 |
| Microbiological data |  |  |  |
| At least two positive blood culture sets, n (%) | 217 (50) | 106 (92) | <0.001 |
| Persistent bacteremia ≥48h, n (%) | 28 (7) | 13 (11) | 0.108 |
| Polymicrobial bacteremia, n (%) | 189 (44) | 11 (10) | <0.001 |
| Setting of infection onset |  |  | <0.001 |
| Community, n (%) | 85 (20) | 61 (53) |  |
| Healthcare-associated, n (%) | 88 (20) | 29 (25) |  |
| Nosocomial, n (%) | 261 (60) | 25 (22) |  |
| Manifestations (within 72h from bacteremia onset) |  |  |  |
| Fever (temperature >38°C), n (%) | 362 (83) | 94 (82) | 0.676 |
| Duration of systemic symptoms (days), median (IQR) | 1 (1-1) | 7 (2-21) | <0.001 |
| Sepsis or septic shock, n (%) | 219 (51) | 44 (38) | 0.021 |
| Cardiac murmur, n (%) | 60 (14) | 78 (68) | <0.001 |
| Embolic events, n (%) | 9 (2) | 54 (47) | <0.001 |
| Cerebral embolic events, n (%) | 5 (1) | 37 (32) | <0.001 |
| Non-cerebral embolic events, n (%) | 6 (1) | 37 (32) | <0.001 |
| Acute native bone and joint infection, n (%) | 13 (3) | 9 (8) | 0.029 |
| Immunologic phenomena^a^, n (%) | 0 (0) | 5 (4) | <0.001 |
| Known origin of infection, n (%) | 306 (71) | 3 (3) | <0.001 |
| Cardiac imaging |  |  |  |
| TTE, n (%) | 183 (42) | 101 (89) | <0.001 |
| TEE, n (%) | 54 (12) | 76 (66) | <0.001 |
| [^18^F]FDG-PET/CT, n (%) | 21 (5) | 40 (35) | 0.021 |
| Cardiac-CT, n (%) | 2 (0.5) | 7 (6) | <0.001 |
| Any cardiac imaging, n (%) | 203 (47) | 115 (100) | <0.001 |
| Laboratory values upon bacteremia onset |  |  |  |
| Hemoglobin (g/dL), median (IQR) | 99 (84-117) | 97 (90-116) | 0.828 |
| Platelet count (G/l), median (IQR) | 220 (138-305) | 199 (137-275) | 0.339 |
| Albumin (g/l)^b^, median (IQR) | 29 (26-34) | 32 (29-36) | 0.050 |
| Outcome |  |  |  |
| Recurrence of *E. faecalis* bacteremia within 90 days, n (%) | 9 (2) | 3 (3) | 1.000 |
| New *E. faecalis* infective endocarditis within 90 days, n (%) | 2 (0.5) | 3 (3) | 0.065 |
| Mortality within 90 days, n (%) | 122 (28) | 20 (17) | 0.023 |

[^18^F]FDG-PET/CT: [^18^F]fluorodeoxyglucose positron emission tomography/CT; IQR: interquartile range; TTE: transthoracic echocardiography; TEE: transesophageal echocardiography

^a^Defined according to the 2023 International Society of Cardiovascular Infectious Diseases Duke criteria

^b^Values for albumin in 280 (51%) episodes.

**Supplementary Table 4.** Diagnoses and cardiac imaging exams of episodes with recurrent *E. faecalis* bacteremia within 90 days of the initial episode

|  | **Initial episode** | | **Subsequent episode** | |
| --- | --- | --- | --- | --- |
|  | **Diagnosis** | **Cardiac imaging** | **Diagnosis** | **Cardiac imaging** |
| 1 | Infective endocarditis | TTE, TEE | Infective endocarditis | TTE, TEE |
| 2 | Infective endocarditis | TTE | Infective endocarditis | TEE, [^18^F]FDG-PET/CT |
| 3 | Infective endocarditis | TTE, TEE, [^18^F]FDG-PET/CT | Infective endocarditis | TTE, TEE, [^18^F]FDG-PET/CT |
| 4 | Unknown origin | TTE, TEE, [^18^F]FDG-PET/CT | Infective endocarditis | TTE, TEE |
| 5 | Unknown origin | TTE, TEE | Infective endocarditis | TTE, TEE, [^18^F]FDG-PET/CT |
| 6 | Unknown origin | TTE, TEE | Unknown origin | TTE, TEE |
| 7 | Urinary-tract | TTE | Spondylodiscitis | TTE, TEE |
| 8 | Urinary-tract |  | Spondylodiscitis | TTE, TEE |
| 9 | Urinary-tract |  | Urinary-tract | TTE |
| 10 | Urinary-tract | TTE | Catheter-related | TTE, TEE |
| 11 | Catheter-related | TTE, TEE, [^18^F]FDG-PET/CT | Unknown origin | TTE, TEE, [^18^F]FDG-PET/CT |
| 12 | Cholangitis |  | Cholangitis | TTE |

[^18^F]FDG-PET/CT: [^18^F]fluorodeoxyglucose positron emission tomography/computed tomography; TEE: transesophageal echocardiography; TTE transthoracic echocardiography
